# Quick Transposition of ReBOSSIS-J® to the Host Bone Trabeculae Within One Month After Supplementing to the Harvest Site on the Calcaneus for Autologous Bone Grafting in a Rheumatoid Arthritis Case

**DOI:** 10.7759/cureus.45812

**Published:** 2023-09-23

**Authors:** Takaaki Noguchi, Makoto Hirao, Gensuke Okamura, Jun Hashimoto

**Affiliations:** 1 Orthopaedic Surgery, National Hospital Organization, Osaka Minami Medical Center, Kawachinagano, JPN

**Keywords:** quick absorption and bone formation, rheumatoid arthritis (ra), foot and ankle surgery, autologous calcaneal bone graft, rebossis-j®

## Abstract

We present the case of a patient with rheumatoid arthritis who underwent talonavicular joint fusion using an autologous calcaneal bone graft. At the same time, the bony defect at the harvest site was supplemented with ReBOSSIS-J^®^ [70% β-TCP and 30% poly(L-lactide-co-glycolide)](ORTHOREBIRTH Co. Ltd., Kanagawa, Japan), a synthetic bioresorbable bone void filler for the repair of bony defects with handling characteristics similar to a cotton ball. Material resorption and new bone formation had already started one week postoperatively. Transposition to host bone trabeculae was almost completed by 26 days postoperatively. Very rapid reactive graft resorption, repair with new bone formation, and subsequently, most of the transformation to host bone trabeculae were confirmed. ReBOSSIS-J^®^ appears feasible to contribute to early heel weight-bearing exercise after foot or ankle surgery. In addition, preventing the fracture at the harvesting site of the calcaneal bone graft can also be expected.

## Introduction

Even under pharmacotherapy against rheumatoid arthritis (RA), situations requiring partial joint-fusion surgery of the foot are often seen. In such situations, a calcaneal bone graft is useful because of the convenience of harvesting the graft from close to the same surgical site in the foot [1]. However, harvest sites with huge bony defects need to be filled with a bone void filler. Beta-tricalcium phosphate (β-TCP) has been reported as a useful material for filling bony defects, with material resorption and new bone formation seen three months after surgery and almost complete transposition of the harvest site to the host bone trabeculae by 12 months after surgery [2]. To further expedite rehabilitation to the point of weight-bearing or heel walking and also to prevent the fracture at the harvesting site of a calcaneal bone graft, earlier transposition to the host bone is preferable because the strength of the harvest site would then be increased or normalized earlier.

In the present case, ReBOSSIS-J® [70% β-TCP and 30% poly(L-lactide-co-glycolide)] (ORTHOREBIRTH Co. Ltd., Kanagawa, Japan), a synthetic bioresorbable bone void filler for the repair of bony defects with handling characteristics of cotton ball-like form [3], was used to be expected to allow earlier transposition to host bone because of its composition, cotton ball-like structure, and softness. In fact, microfiber meshes of the silicon content in ReBOSSIS-J® have been reported to stimulate osteogenic cells to mineralize and form bone [3-7]. Furthermore, the cotton ball-like structure provides a scaffold for osteoclast/osteoblast adhesion and supports osseous tissue in crossing bony defects [3-7]. In this study, a new synthetic bioresorbable bone void filler, ReBOSSIS-J®, was utilized for the harvesting site of calcaneal trabecular bone grafts.

## Case presentation

**Figure 1:**
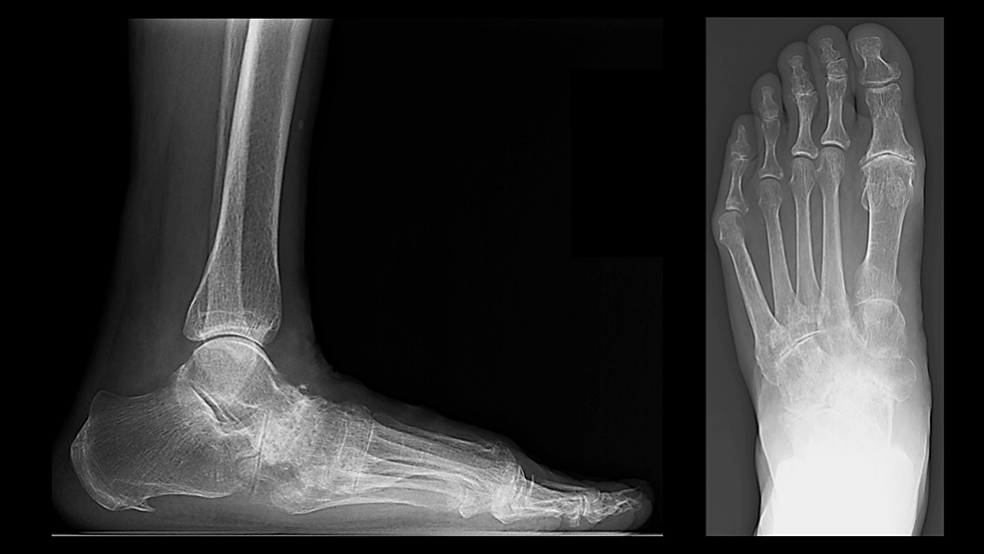
Preoperative radiograph of the foot in a weight-bearing position. The talonavicular joint is destroyed and shows end-stage arthritis. The talus shows a plantar flexion deformity.

A 74-year-old woman with RA (disease duration: 25 years) presented with painful talonavicular joint destruction (Figure 1) even under pharmacotherapy with 8mg of methotrexate administration.

**Figure 2:**
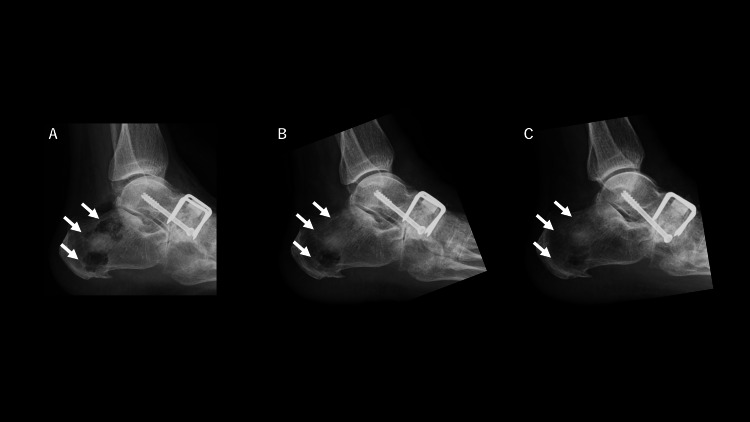
Postoperative radiograph of the foot (~14 days) (A) Just after surgery, Day 0: Talonavicular joint fusion is complete. The bony defect at the harvest site is supplemented with ReBOSSIS-J®. Three white arrows indicate the site of calcaneal bone harvesting. The central part was especially filled with ReBOSSIS-J®. The superior and inferior parts seemed to be less filled with material. (B) After one week, material resorption and new bone formation had already started and progressed two weeks after surgery (C). Three white arrows indicate the site of calcaneal bone harvesting. Not only the central part but also the superior and inferior parts were also filled with the newly formed bone.

As a condition of disease control, the Disease Activity Score 28 (DAS-28-CRP) showed 2.52, suggesting good disease control. However, joint destruction or deformity in her foot progressed. Conservative treatments, including non-steroidal anti-inflammatory drugs and wearing an insole, failed to reduce pain. She was referred to our institution for corrective surgery and underwent a talonavicular joint fusion with an autologous calcaneal bone graft, dense hydroxyapatite, staples, and screws. At the same time, the bony defect at the harvest site was supplemented with ReBOSSIS-J® (Figure 2A).

**Figure 3:**
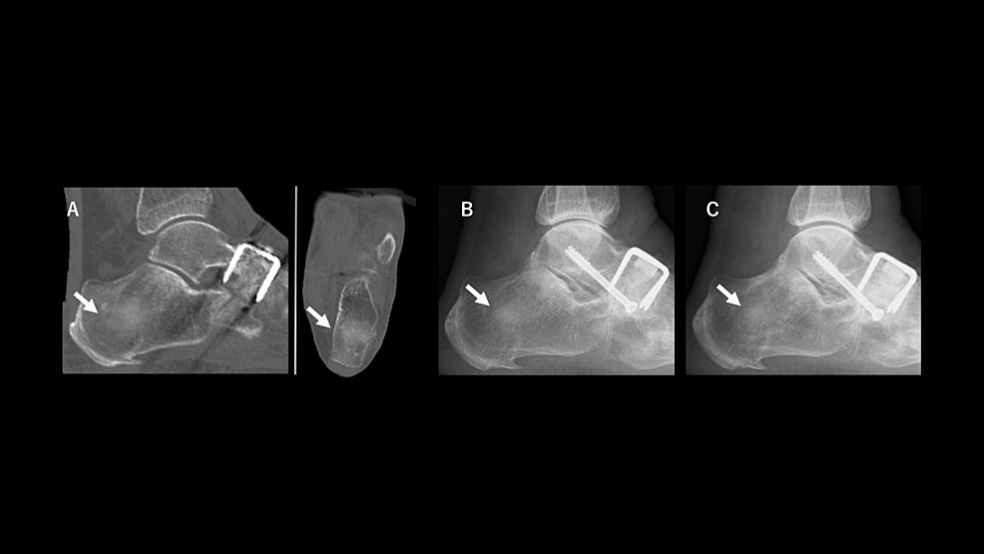
Postoperative radiograph and computed tomography (CT) of the foot (26 days~) (A): Sagittal and transverse views of CT analysis at 26 days after surgery. Transposition to host bone trabeculae was almost complete. Talonavicular joint fusion also seems to be complete. The white arrow indicates the site of calcaneal bone harvesting, filled with newly formed bone. (B), (C): There is no problem at the harvesting site one month (B) and three months (C) after surgery. The talus dorsiflexion correction was completed. The white arrow indicates the site of calcaneal bone harvesting, filled with newly formed bone, and continuation with the host bone trabeculae was also observed.

Material resorption and new bone formation were seen to have already started one to two weeks after surgery (Figures 2B, 2C). Subsequent transposition to host bone trabeculae was almost completed by 26 days after the surgery (Figure 3A).

No problems at the harvest site or fusion at the talonavicular joint were seen at one and three months after surgery (Figures 3B, 3C). Very rapid reactive graft resorption, new bone formation and repair, and subsequently most of the transformation to host bone were confirmed. Full weight-bearing and walking were therefore introduced four weeks postoperatively. Five weeks after surgery, the patient was able to leave the hospital in an ambulatory state without needing a cane. As of the time of writing, the patient can walk for over an hour and can also jog. Clinical scores such as the Japanese Society for Surgery of the Foot RA foot score [8, 9] were significantly improved from 55 to 87 points (full score: 100).

## Discussion

Although this case report involves only a single case, filling ReBOSSIS-J® into the site from which the autologous bone graft was harvested in the calcaneus showed astonishingly quick transposition to host bone trabeculae. Very rapid processes of reactive graft resorption, new bone formation, repair, and subsequently most of the transformation to host bone were confirmed. These phenomena have the possibility of contributing to expediting heel walking exercises after foot or ankle reconstruction surgery using an autologous calcaneal bone graft. Weight-bearing and gait exercises in the early postoperative phase are important from the perspective of preventing osteopenia resulting from prolonged bedrest, disuse muscle atrophy, weakness, and subsequent decreases in physical mobility [10-16]. ReBOSSIS-J® might prove extremely useful as a bone void filler for sites from which autologous bone grafts are harvested from the calcaneus. From the perspective of preventing the fracture at the harvesting site of a calcaneal bone graft, ReBOSSIS-J® also might be useful. The composition of synthetic materials and/or the characteristics of their cotton ball-like form are considered advantageous in promoting bone metabolism and transposition. Innovations in postoperative procedures for rehabilitation after foot reconstructive surgery using calcaneal autologous bone grafts can be expected. In addition, preventing the fracture at the harvesting site of the calcaneal bone graft can also be expected. The limitation of this study is that there is no comparative data; however, conventional granular β-TCP showed transposition within three to 12 months in our previous experience [2], so the characteristics of ReBOSSIS-J® should have an advantage for rapid transposition. As future research directions, further investigations of ReBOSSIS-J® with increased numbers of cases are warranted.

## Conclusions

In conclusion, a new synthetic bioresorbable bone void filler, ReBOSSIS-J®, showed very rapid reactive graft resorption, repair with new bone formation, and subsequently, most of the transformation to host bone trabeculae were confirmed at the calcaneal bone grafting site within one month. ReBOSSIS-J® appears feasible to contribute to early heel weight-bearing exercise after foot or ankle surgery. In addition, preventing the fracture at the harvesting site of the calcaneal bone graft can also be expected.
